# MAGC-DTI: modality-shared space and adaptive gated interactive cross-attention for drug–target interaction prediction

**DOI:** 10.1038/s41598-026-50141-w

**Published:** 2026-06-04

**Authors:** Bowen Wang, Xiaolan Xie, Haitao Zou, Shaoliang Peng

**Affiliations:** 1 College of Computer Science and Engineering, Guilin University of Technology, Guilin, 541006 Guangxi China; 2 College of Computer Science and Electronic Engineering, Hunan University, Changsha, 410082 Hunan China

**Keywords:** Drug-target interaction, Deep learning, Cross-modal learning, Multi-scale features, Cold-start prediction, Cross-domain generalization, Computational biology and bioinformatics, Drug discovery

## Abstract

Accurate drug–target interaction (DTI) prediction is crucial for drug repurposing and accelerating drug development. Although deep learning has advanced DTI prediction, existing methods struggle with two key challenges: (i) capturing complex hierarchical patterns in protein sequences, and (ii) enabling effective bidirectional information exchange between drug and protein modalities. We propose MAGC-DTI, an end-to-end cross-modal framework that integrates bidirectional information exchange into both feature extraction and fusion stages through three key innovations: (i) multi-scale attention aggregation (MSAA) for hierarchical protein pattern capture, (ii) adaptive gated interactive cross attention (AGICA) for context-aware cross-modal interaction, and (iii) multi-path residual classifier (MPRC) for modality-preserving fusion. Comprehensive evaluations on six benchmark datasets show that MAGC-DTI generally achieves favorable performance relative to seven state-of-the-art baselines, with competitive results in cold-start and cross-domain scenarios. The model also provides interpretable insights through attention visualization and case studies confirm the biological relevance of learned representations.

## Introduction

Accurate identification of drug-target interactions (DTIs) is fundamental to drug discovery and repurposing. Since traditional experimental assays are time-consuming and costly^1–3^, computational approaches have become essential for efficiently screening large chemical libraries^4–10^. While recent advances in deep learning have significantly improved prediction accuracy^11–13^, developing models that generalize well to novel compounds remains a critical challenge.

Existing computational methods broadly fall into three categories: docking-based, network-based, and machine learning-based approaches. Molecular docking offers mechanistic insights but relies heavily on high-quality 3D structures, which are unavailable for many targets^14–18^. Network-based algorithms exploit graph topology and relational structure to infer biomolecular associations, but often struggle with ”cold-start” scenarios, failing to predict interactions for new drugs or targets absent from the training network^19–22^. Deep learning methods, particularly those integrating graph representation learning, attention mechanisms, and neural architectures for drug discovery-related prediction tasks, have enabled automated feature extraction and improved predictive performance^23–29^. However, most current sequence-based models suffer from two key limitations: (i) they often treat drug and protein feature extraction as independent processes, neglecting the dynamic, bidirectional structural adaptations inherent in molecular binding; and (ii) they fail to capture the hierarchical structural patterns of proteins effectively. Furthermore, many models exhibit severe performance degradation in cross-domain or cold-start settings due to distribution shifts^30^.

To address these challenges, we propose MAGC-DTI, an end-to-end cross-modal framework that integrates bidirectional information exchange into both feature extraction and fusion stages. Our contributions are threefold: (i) a Multi-Scale Attention Aggregation (MSAA) module that performs selective multi-scale alignment of protein representations to capture hierarchical patterns; (ii) an Adaptive Gated Interactive Cross Attention (AGICA) module that conducts bidirectional cross-modal interaction in a shared space, utilizing an order-sensitive cascade (MSAA *→* AGICA) to enhance information flow; and (iii) a Multi-Path Residual Classifier (MPRC) that preserves modality-specific information via dual-path residual processing. Coupled with conditional adversarial domain adaptation (CDAN) for cross-domain scenarios, MAGC-DTI shows improved generalization ability under challenging settings.

We evaluate MAGC-DTI on six benchmark datasets under diverse settings, including random splits, cold-start, unseen drug/target scenarios, and clustering-based cross-domain evaluation. Coupled with conditional adversarial domain adaptation (CDAN) for cross-domain scenarios, MAGC-DTI shows cross-domain generalization capability. Attention visualization and case studies further suggest that the model can capture biologically meaningful interaction patterns.

## Results

### Baseline methods

We compare MAGC-DTI with several representative and recent DTI prediction models. MolTrans ^31^ decomposes drug and protein inputs into substructure sequences and models their interactions using Transformer-based modules. DrugBAN ^32^ employs a bilinear attention network to capture local drug–target interactions and further incorporates conditional domain adaptation for cross-domain prediction. BINDTI ^33^ introduces a bi-directional Intention network for bidirectional drug–protein feature interaction. MADTI ^34^ implements a multi-domain awareness scheme. WDGBANDTI ^35^ combines weighted deep graph convolution with bilinear attention. ASCS-DTI ^36^ is a recent multi-modal DTI framework that integrates substructure attention, DMPNN-based molecular topology modeling, molecular fingerprint information, and feature interaction fusion. CF-DTI ^37^ is a recent coarse-to-fine DTI framework that combines focal/cross-attention, multi-granularity interaction learning, and adaptive fusion learning. These methods were selected because they provide strong and methodologically relevant baselines for evaluating MAGC-DTI. In particular, they cover several important directions in recent DTI research, including Transformer-based interaction modeling, bilinear attention, bidirectional interaction learning, substructure-aware molecular representation, and coarse-to-fine multi-granularity fusion. Therefore, comparing MAGC-DTI with these methods enables a more informative assessment of its performance relative to recent representative DTI prediction frameworks.

### Performance comparison

To comprehensively evaluate the performance of MAGC-DTI, we conducted extensive experiments following three data partitioning strategies (S1–S3) plus a clustering-based evaluation approach, and compared our model with seven state-of-the-art baseline methods. This section presents detailed experimental results.

#### Performance under random split (S1)

To establish a baseline evaluation, we implemented a random split strategy (S1) by dividing each dataset into training, validation, and test sets at a ratio of 7:1:2. Tables 1 and 2 present comprehensive performance comparisons between MAGC-DTI and baseline methods across six benchmark datasets.

**Table 1:** Performance comparison on BindingDB, BioSNAP, and DrugBank under S1.

Method	BindingDB		BioSNAP		DrugBank	
	AUROC	AUPRC	AUROC	AUPRC	AUROC	AUPRC
MolTrans	0.9354±0.0033	0.9084±0.0044	0.8796±0.0060	0.8829±0.0071	0.8600±0.0049	0.8360±0.0020
Drug-BAN	0.9600±0.0010	0.9480±0.0020	0.9030±0.0050	0.9020±0.0040	0.8813±0.0010	0.8740±0.0020
BINDTI	0.9607±0.0019	0.9446±0.0024	0.9039±0.0044	0.9020±0.0065	0.8819±0.0038	0.8830±0.0044
MADTI	0.9660±0.0010	0.9540±0.0010	0.9040±0.0020	0.9116±0.0030	0.8706±0.0030	0.8749±0.0050
WDGBANDTI	0.9607±0.0010	0.9491±0.0020	0.9140±0.0030	0.9250±0.0020	0.8857±0.0020	0.8848±0.0030
ASCS-DTI	0.9650±0.0040	0.9500±0.0070	0.9164±0.0050	0.9172±0.0080	0.8831±0.0020	0.8865±0.0030
**MAGC-DTI**	**0.9701±0.0027**	**0.9613±0.0010**	**0.9286±0.0023**	**0.9350±0.0040**	**0.9075±0.0010**	**0.9147±0.0020**

The best values are shown in bold.

**Table 2:** Performance comparison on human, KIBA, and *C. elegans* under S1.

Method	Human		KIBA		*C. elegans*	
	AUROC	AUPRC	AUROC	AUPRC	AUROC	AUPRC
MolTrans	0.9750±0.0040	0.9770±0.0030	0.8120±0.0010	0.7884±0.0010	0.9870±0.0030	0.9890±0.0020
Drug-BAN	0.9831±0.0030	0.9773±0.0040	0.8922±0.0060	0.7222±0.0035	0.9887±0.0030	0.9903±0.0020
BINDTI	0.9813±0.0040	0.9769±0.0045	0.9140±0.0025	0.7884±0.0010	0.9873±0.0020	0.9895±0.0010
MADTI	0.9830±0.0030	0.9768±0.0030	0.8922±0.0060	0.7222±0.0035	0.9840±0.0025	0.9852±0.0030
WDGBANDTI	0.9823±0.0012	0.9753±0.0030	0.9234±0.0018	0.7834±0.0020	0.9916±0.0010	0.9925±0.0014
ASCS-DTI	0.9840±0.0040	0.9825±0.0050	0.9232±0.0019	0.7954±0.0023	0.9890±0.0030	0.9920±0.0030
CF-DTI	0.9872±0.0020	0.9834±0.0025	0.9293±0.0023	0.8012±0.0020	0.9926±0.0020	0.9928±0.0020
**MAGC-DTI**	**0.9914±0.0009**	**0.9878±0.0009**	**0.9365±0.0046**	**0.8213±0.0014**	**0.9960±0.0012**	**0.9966±0.0005**

The best values are shown in bold.

Under the random split setting, MAGC-DTI achieves the best overall performance among the compared methods across the six benchmark datasets. In terms of AUROC, relative to the strongest baseline on each dataset, MAGC-DTI shows improvements ranging from 0.34% on C.elegans to 2.43% on DrugBank, suggesting favorable and consistent predictive performance across datasets with different characteristics. Similar trends are observed for AUPRC, where the relative improvements range from 0.38% to 2.67%. In addition, MAGC-DTI exhibits relatively small standard deviations on most datasets, further supporting the stability and reliability of its performance under this setting. While the default MAGC-DTI framework demonstrates strong performance using its custom GCN and MSAA encoders, its core advantage also lies in the robust cross-modal interaction enabled by the Adaptive Gated Interactive Cross Attention (AGICA) and downstream fusion modules. To evaluate the framework’s extensibility and compatibility with advanced feature representations, we replaced the default drug and target encoders with pretrained ChemBERTa ^38^ and ESM-2 ^39^ models, respectively (denoted as MAGC-DTI+PLMs). As shown in Supplementary Table S2, this enhancement led to consistent improvements on both DrugBank and BioSNAP. Specifically, the AUROC and AUPRC increased by 3.27% and 2.42% on DrugBank, and by 1.33% and 0.59% on BioSNAP, respectively. These results indicate that the downstream interaction and fusion mechanisms of MAGC-DTI can seamlessly integrate and benefit from richer token-level representations, underscoring its broad applicability in drug-target interaction prediction.

#### Performance under 5-fold cross-validation (S2)

To further assess model stability and reduce the bias introduced by a single train–test partition, we conducted 5-fold cross-validation (S2), in which each dataset was divided into five equal subsets, with each subset serving as the test set once while the remaining four subsets were used for training. This evaluation strategy provides a more reliable estimate of model performance across different data folds. Supplementary Tables S3 and S4 summarize the AUROC and AUPRC results of all compared methods under this setting.

Evaluation across six benchmark datasets using 5-fold cross-validation indicates that MAGC-DTI performs competitively. Specifically, the model shows favorable results on DrugBank, Human, and KIBA, while maintaining stable performance on BindingDB, BioSNAP, and C.elegans. These observations suggest that the model’s effectiveness is relatively robust to different data partitions rather than being sensitive to specific folds.

To further assess the statistical significance of the performance differences, we conducted t-tests based on the AUROC values between MAGC-DTI and four baseline models under both the S1 and 5-fold cross-validation settings (Supplementary Table S5). The results demonstrate that MAGC-DTI achieves statistically significant improvements (*p < 0.05*) on BioSNAP, DrugBank, KIBA, and C.elegans across both evaluation protocols. On BindingDB and Human, the performance of MAGC-DTI was numerically comparable to or marginally higher than the baselines, though the differences did not consistently reach statistical significance under the 5-fold cross-validation setting.

#### Performance under cold pair split (S3)

**Fig. 1:**
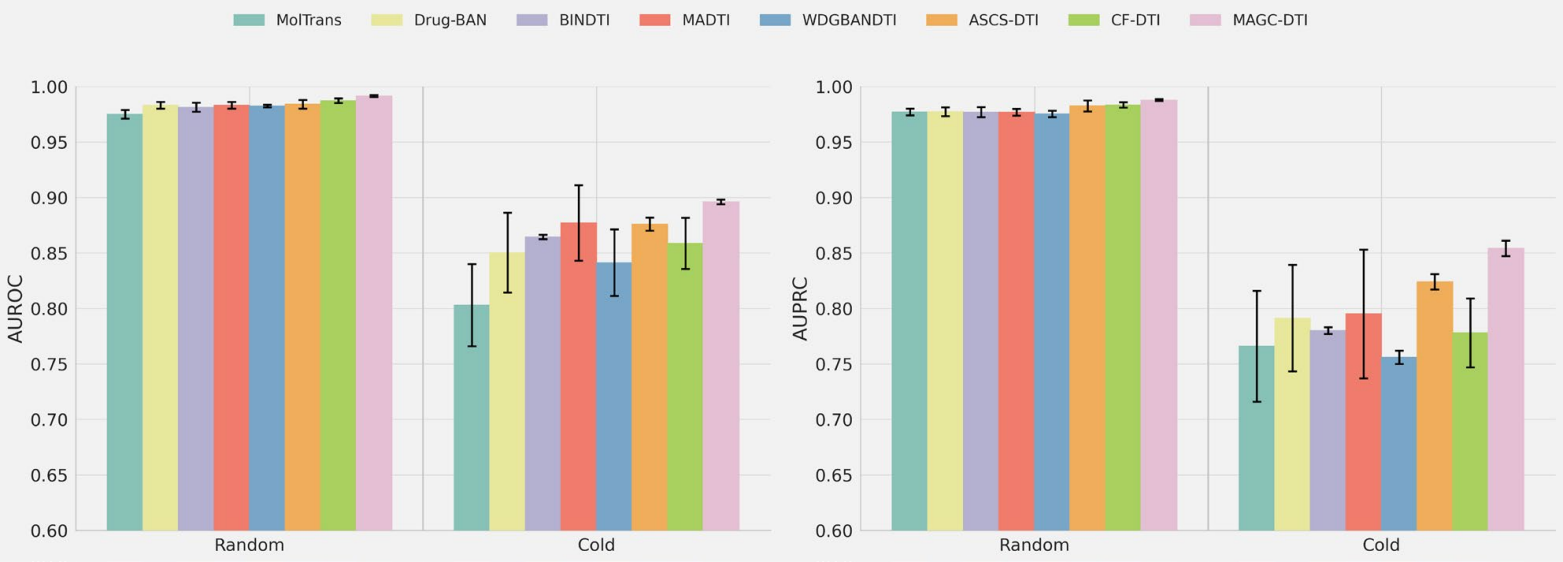
Performance comparison between random split (S1) and cold pair split (S3) on the Human dataset. Left: AUROC; Right: AUPRC. Bars show mean values across runs and error bars denote standard deviation (s.d.). The cold pair split excludes specific drug–target pairs from training to evaluate prediction of novel combinations (see “Methods”).

Predicting interactions between novel drug-target combinations represents a significant challenge in DTI modeling. To evaluate this capability, we conducted experiments under the cold pair split strategy, where specific drug-target pairs in the test set never appear in the training set, though the individual drugs and targets may be present in different combinations. Figure 1 illustrates the performance comparison between random split (S1) and cold pair split (S3) on the Human dataset.

Under random split conditions, all deep learning methods achieved high performance metrics, with AUROC values consistently exceeding 0.97. However, as noted by Chen et al.^40^, the Human dataset contains hidden ligand bias, causing models to potentially rely on drug features rather than genuine interaction patterns. This bias explains the artificially inflated performance observed under random split conditions.

The cold pair split results in Fig. 1 reveal a more realistic assessment of model capabilities. All methods experienced significant performance decreases, with MolTrans showing the most dramatic decline, AUROC decreasing by approximately 17.2%. The AUPRC metrics exhibited similar trends, with substantial decreases across all methods. Despite this difficulty, MAGC-DTI still achieves the best overall performance under the cold-pair setting. Compared with the strongest baseline, MAGC-DTI improves AUROC by approximately 2.18% and AUPRC by approximately 3.65%. Additionally, MAGC-DTI showed smaller variance compared to competing methods.

The substantial performance gap between random and cold pair splits highlights the inherent difficulty of predicting novel interaction combinations. The results under the cold-pair setting suggest that MAGC-DTI may capture interaction patterns that transfer better to unseen drug-target combinations. This property could be useful in settings where novel combinations need to be screened.

#### Performance under unseen drug and unseen target settings (S4)

We further evaluated MAGC-DTI under the unseen drug and unseen target settings on the BioSNAP dataset to assess its entity-level cold-start generalization. As these settings require the model to generalize to previously unseen drugs or targets, they are more challenging than the cold-pair split. Supplementary Table S6 summarizes the corresponding AUROC results.

As expected, all methods exhibit further performance degradation under these stricter settings, particularly in the unseen target setting. This suggests that generalizing to unseen proteins is generally more difficult than predicting interactions for unseen drugs, likely because target representations are harder to transfer across structurally and functionally diverse proteins.

Under these more challenging settings, MAGC-DTI achieves the best performance in both cases. Compared with the strongest baselines, it improves AUROC by 0.52% and 0.49% in the unseen drug and unseen target settings, respectively. Although these gains are relatively modest, they still suggest that MAGC-DTI maintains reasonable generalization ability under stricter entity-level cold-start evaluation.

Overall, these findings provide additional evidence that MAGC-DTI generalizes reasonably well when either drugs or targets are not observed during training.

#### Performance under clustering-based segmentation strategy

Cross-domain testing, where the test set comprises both unseen samples and data outside the learned distribution, presents the most challenging evaluation. We adopted the cross-domain setup from Bai et al. (DrugBAN)^32^: drugs and proteins were clustered using single-linkage on ECFP4 fingerprints and pseudo amino acid composition (PSC)^41^; 60% of drug clusters and 60% of protein clusters were randomly assigned to the source domain and the rest to the target domain. All pairwise combinations between source-domain drugs and proteins formed the training set, while target-domain pairs were used for testing.

**Fig. 2:**
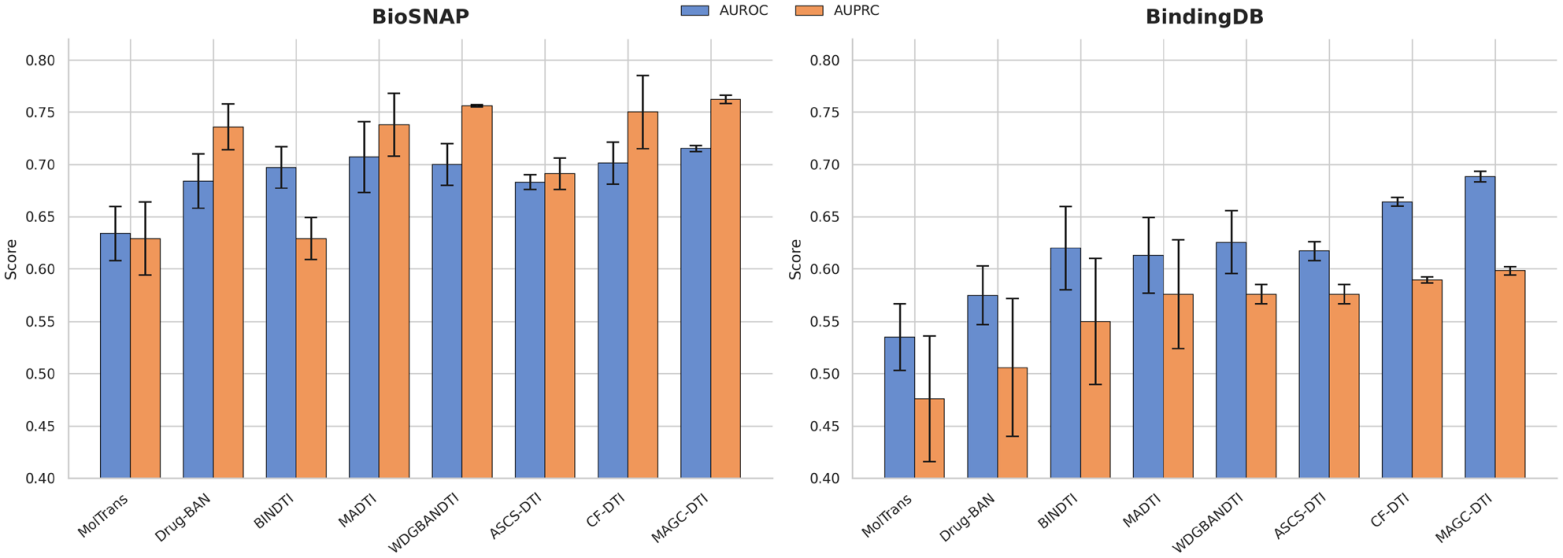
Cross-domain performance under the clustering-based pair split. Left: BioSNAP; Right: BindingDB. For each method, blue bars denote AUROC and orange bars denote AUPRC; error bars indicate standard deviation across runs.

Figure 2 summarizes cross-domain performance. MAGC-DTI achieves the best overall results among the compared methods. On BioSNAP, its improvements over the next-best performer are modest, and on BindingDB, it also ranks first across the reported metrics.

These results indicate that MAGC-DTI generalizes well to structurally distinct domains; we attribute this robustness to the multi-scale attention aggregation and adaptive gated cross-attention modules, which learn domain-robust representations that transfer across chemical spaces.

### Ablation study

To comprehensively evaluate the contribution of each component in MAGC-DTI, we conducted ablation and variant analysis experiments on multiple benchmark datasets. The main ablation study focused on four core components: (1) the Adaptive Gated Interactive Cross Attention (AGICA) module; (2) the Multi-Scale Attention Aggregation (MSAA) mechanism; (3) the Multi-Path Residual Classifier (MPRC); and (4) the Graph Convolutional Network (GCN)-based drug encoder. We further examined the contribution of CDAN under the cross-domain setting.

#### Effect of adaptive gated interactive cross attention

To assess the effectiveness of the AGICA module, we replaced it with a simple concatenation operation (MAGC-DTI w/o AGICA) while keeping all other components unchanged. As shown in Supplementary Table S7, removing AGICA results in a consistent performance drop across all datasets. Specifically, the decrease in AUROC ranges from 0.5% to 4.0%, and the decrease in AUPRC ranges from 0.4 to 4.0%. The most significant drops are observed on DrugBank (AUROC: 4.0%, AUPRC: 4.0%), while the performance on *C. elegans* is least affected. These results indicate that AGICA plays a central role in modeling cross-modal interactions between drug and protein features, thereby improving DTI prediction accuracy.

#### Effect of multi-scale attention aggregation

To evaluate the contribution of the MSAA mechanism, we constructed a variant (MAGC-DTI w/o MSAA) by replacing it with a standard feature fusion approach. As shown in Supplementary Table S8, removing MSAA leads to a noticeable performance decline, with the largest decreases observed on DrugBank (AUROC: 2.2%, AUPRC: 3.7%) and BioSNAP (AUROC: 2.2%, AUPRC: 2.4%). These results suggest that MSAA is important for capturing protein sequence patterns at multiple receptive-field scales, which in turn improves downstream interaction modeling.

#### Effect of multi-path residual classifier

We further investigated the importance of the MPRC module by replacing it with a standard MLP classifier (MAGC-DTI w/o MPRC). As shown in Supplementary Table S9, the multi-path residual architecture performs better than the single-path alternative across all reported datasets and metrics. The performance drop is most pronounced on DrugBank (AUROC: 2.1%, AUPRC: 2.0%) and BioSNAP (AUROC: 1.7%, AUPRC: 1.6%). These results suggest that the multi-path residual design helps retain complementary modality-specific information while supporting more effective feature fusion.

#### Effect of graph convolutional network module

To evaluate the contribution of the GCN-based drug encoder, we replaced it with a Graph Attention Network (GAT) module (MAGC-DTI w/o GCN). As shown in Supplementary Table S10, this replacement leads to noticeable performance degradation across all datasets. The most significant drops are observed on DrugBank (AUROC: 1.9%, AUPRC: 1.9%) and BioSNAP (AUROC: 1.4%, AUPRC: 1.8%). These results suggest that the GCN-based drug encoder provides a more suitable structural representation than the GAT alternative in our framework, thereby supporting accurate DTI prediction.

#### Contribution of CDAN under the cross-domain setting

To further evaluate the contribution of CDAN under the cross-domain setting, we removed CDAN from MAGC-DTI and compared the resulting variant with the full model on BindingDB and BioSNAP. As shown in Supplementary Table S11, removing CDAN consistently degrades performance on both datasets. In particular, on BindingDB, AUROC and AUPRC decrease by 5.15% and 3.01%, respectively, while on BioSNAP, the corresponding decreases are 1.66% and 2.93%. These results demonstrate that CDAN plays an important role in reducing domain shift and improving the robustness of MAGC-DTI under cross-domain evaluation.

**Fig. 3:**
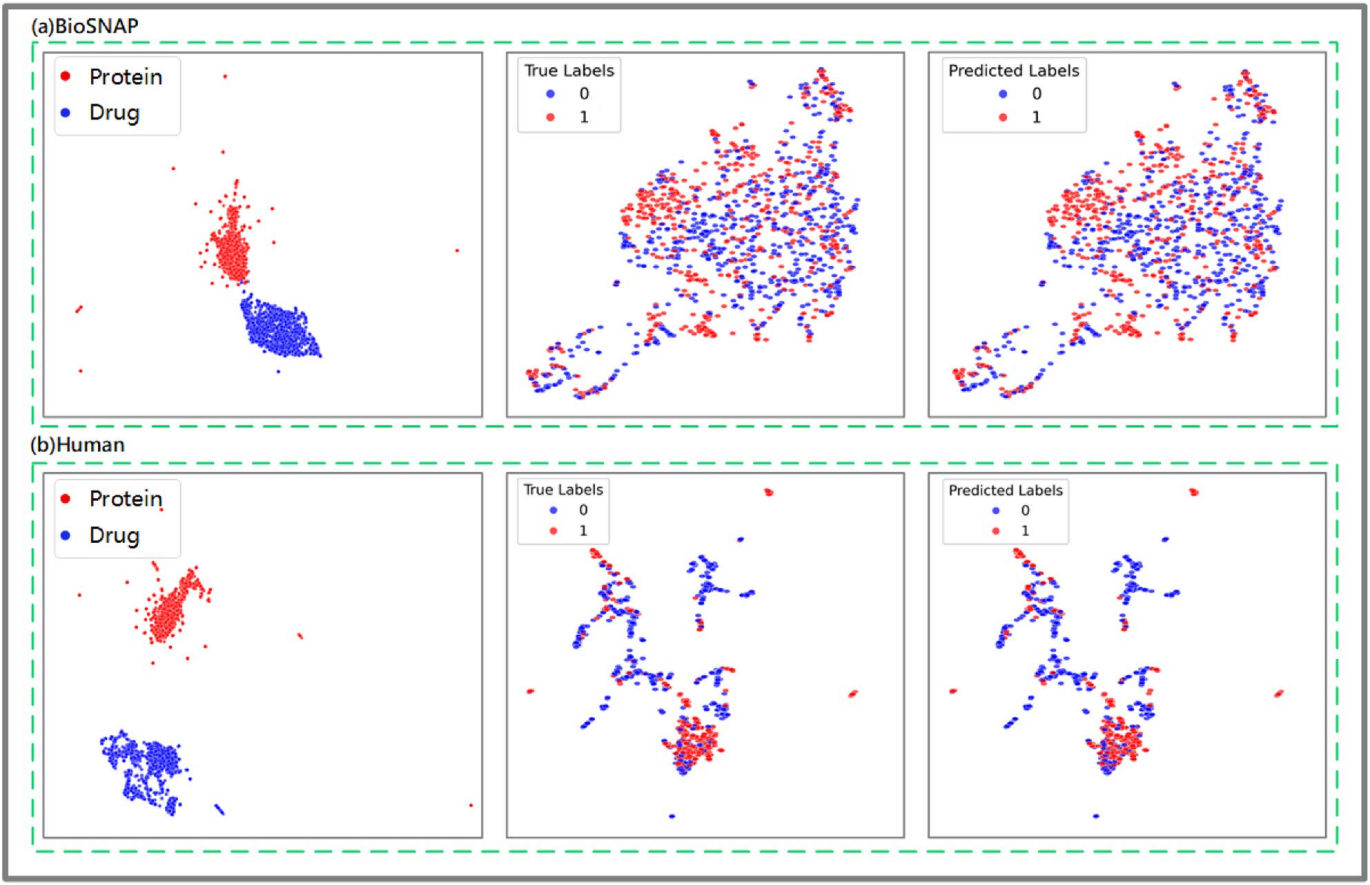
UMAP^42^ feature visualization for BioSNAP and Human test sets. (**a**) BioSNAP; (**b**) human. For each dataset, the three subpanels show (left) fused drug/protein embeddings, (center) joint embeddings colored by true labels, and (right) joint embeddings colored by predicted labels. Colors: blue = non-interacting pairs, red = interacting pairs.

In summary, all components contribute positively to MAGC-DTI, although their relative importance varies across evaluation settings. Under the random-split setting, AGICA has the largest overall impact, with average AUROC and AUPRC drops of approximately 1.63% and 1.87% after removal, and especially large degradation on DrugBank (4.0%/4.0%) and BioSNAP (2.8%/2.8%). MSAA provides the second-largest contribution, particularly on DrugBank and BioSNAP, suggesting that multi-scale protein feature extraction is important for robust representation learning. MPRC and the GCN-based drug encoder also yield consistent gains across datasets, supporting their roles in preserving complementary fused information and modeling molecular topology, respectively. Under the cross-domain setting, CDAN becomes the most critical component, as removing it causes substantial degradation on both BindingDB and BioSNAP, highlighting the importance of domain adaptation for cross-domain generalization (Fig. 3).

### Case studies

To evaluate MAGC-DTI’s capability in identifying novel drug-target interactions beyond benchmark datasets, we conducted comprehensive case studies from both drug-centric and target-centric perspectives. We trained MAGC-DTI on the DrugBank dataset with all existing interactions involving the query drug/target systematically removed from the training set, simulating cold-start scenarios.

For the drug-centric analysis, we selected two biologically significant compounds: NADH (DB00157) and Beta-D-Glucose (DB02379). NADH is a critical coenzyme involved in cellular energy production and has been investigated for its potential therapeutic effects in fatigue-related disorders and cognitive enhancement^43–45^. Beta-D-Glucose serves as the primary energy source for most organisms and plays fundamental roles in numerous biological processes^43^. Table 3 presents the top 10 protein targets predicted to interact with these two compounds. All predicted interactions for NADH achieved prediction scores exceeding 0.9970, while Beta-D-Glucose predictions consistently scored above 0.9990. Of particular note, all interactions except one (P04745 with Beta-D-Glucose marked as unconfirmed) have been experimentally validated according to the DrugBank database, suggesting that MAGC-DTI can prioritize plausible drug-target pairs effectively in this case.

**Table 3:** Top 10 targets predicted to interact with NADH and beta-D-glucose by MAGC-DTI.

Rank	NADH (DB00157)		Beta-D-glucose (DB02379)	
	UniProt ID	Score	UniProt ID	Score
1	O43678	0.9996	P19961	0.9999
2	P37059	0.9991	P04746	0.9999
3	P04035	0.9991	P04745$^*$	0.9999
4	P11586	0.9989	O82839	0.9999
5	Q02338	0.9986	P36924	0.9998
6	P51970	0.9981	P00691	0.9998
7	P37058	0.9980	Q60053	0.9997
8	O95167	0.9976	O85465	0.9996
9	P30043	0.9975	P19571	0.9996
10	P47895	0.9974	Q9WXN1	0.9995

$$^*$$Unconfirmed interaction.

For the target-centric analysis, we selected two pharmacologically important protein targets: histamine H1 receptor (H1R, P35367) and alpha-1C adrenergic receptor (ADRA1C, P35348). H1R plays a crucial role in allergic responses and serves as a target for antihistamine medications, while also mediating neurotransmission in the central nervous system^43,46^. ADRA1C belongs to the G protein-coupled receptor family and is involved in sympathetic nervous system signaling, making it a target for various cardiovascular medications^47^. As shown in Supplementary Table S12, MAGC-DTI accurately identified established drug interactions for both targets. The prediction scores were high, all exceeding 0.9999, indicating the model’s confidence in these predictions. Validation against DrugBank confirmed that all predicted interactions represent true biological relationships.

These case studies suggest that MAGC-DTI can recover several biologically relevant drug-target associations and may provide useful support for drug discovery and repurposing analyses. The predictions may also help narrow the candidate space for subsequent experimental validation.

**Fig. 4:**
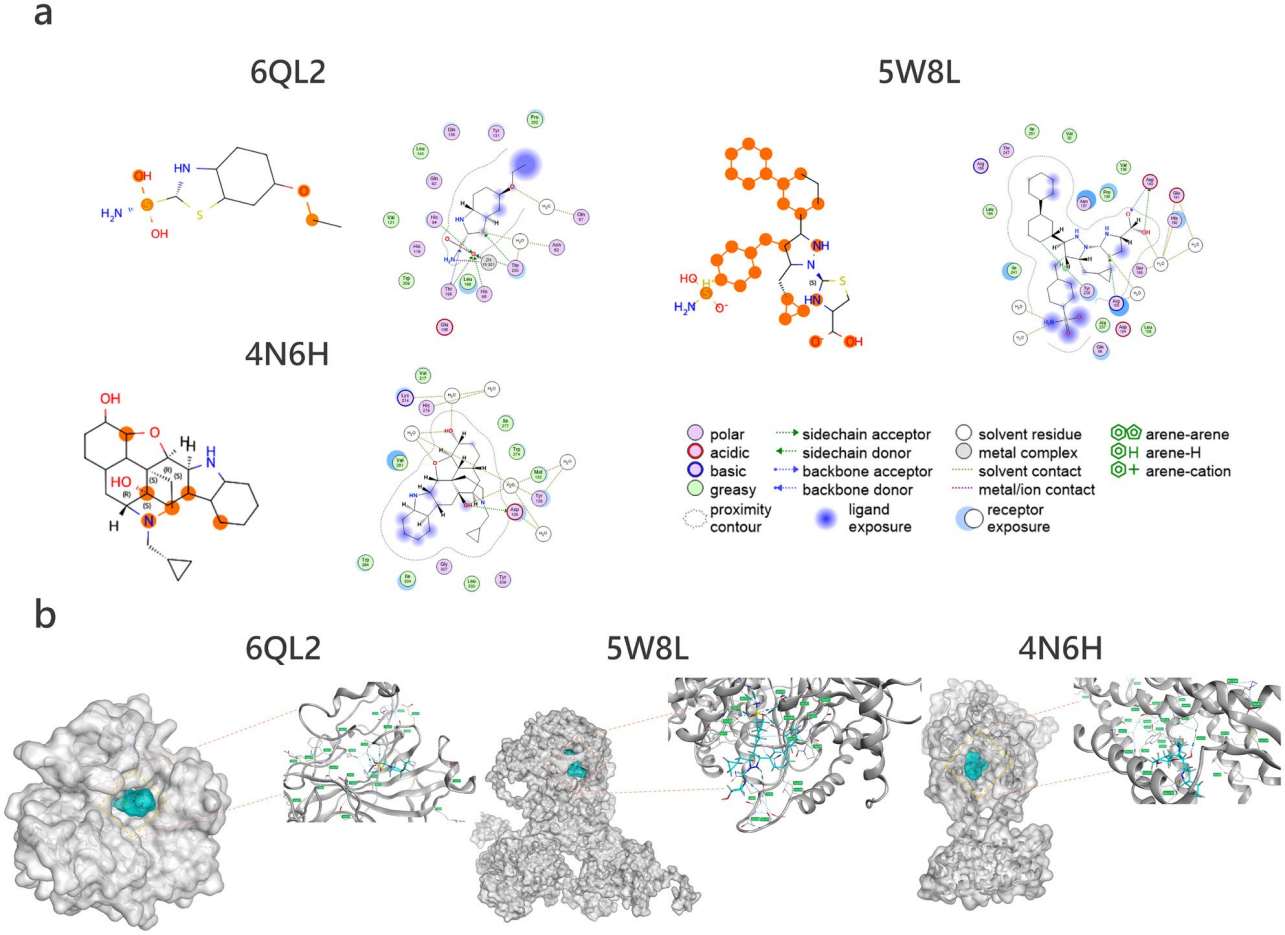
Interpretability visualizations for selected PDB cases. (**a**) 2D ligand depictions with atoms highlighted according to model-derived importance scores (top 10% shown in orange); (**b**) ligand–protein interaction maps and 3D pocket visualizations with residues highlighted according to importance. Attention-derived scores were computed from model attention maps (see “Methods”). 2D ligand images were generated with RDKit^48^; 3D visualizations and interaction maps were produced with MOE^49^.

### Interpretability analysis of cross-bidirectional information exchange

MAGC-DTI offers the advantage of helping researchers understand the reasoning behind its predictions at the molecular level, which is especially valuable for drug development. In this study, we used the attention scores generated by the AGICA (Adaptive Gated Interactive Cross Attention) module of our model. The AGICA module applies a multi-head cross-attention mechanism, where drug and protein features interact deeply through attention. Specifically, the model calculates attention scores by treating drug features as queries and protein features as keys and values, and vice versa. These attention scores reflect how much each atom in the drug or each amino acid in the protein contributes to the final prediction.

We selected three representative cases from the Protein Data Bank (PDB)^50^: 6QL2^51^, 5W8L^52^, and 4N6H^53^. Only high-quality X-ray structures (resolution finer than 2.5Å) were included, and the chosen ligands demonstrated strong binding (pIC_50_ *łe* 100 nM). Importantly, none of these protein-ligand complexes were included in the training, validation, or test sets. Instead, we used a model pre-trained on the DrugBank dataset, and set it to interpretability mode to extract these attention scores for external cases. For visualization, we highlighted the top 10% of atoms or amino acids with the highest attention scores in orange, as shown in Fig. 4.

For PDB structure 6QL2 (ethoxzolamide complexed with human carbonic anhydrase 2), our model successfully identified the sulfonamide region as essential for ligand-protein binding, which is fully consistent with the experimental structure. In the crystal structure, the sulfonamide oxygen acts as a hydrogen bond acceptor to the backbone of Threonine 199, while the amino group serves as a hydrogen bond donor to the side chains of Histidine 94 and Threonine 199. Additionally, the model correctly highlighted the oxygen atom of the ethoxy group as an important interaction point, with experimental data showing this region forms indirect interactions with Glutamine 67 through water molecules. For the benzothiazole scaffold, the model appropriately did not mark it as a critical interaction area, which aligns with observations from the experimental structure where this region primarily contributes through non-specific hydrophobic interactions. Overall, the atoms highlighted by our model correspond to key interaction sites observed in the crystal structure, indicating accurate predictions.

For 5W8L (9YA ligand bound to human L-lactate dehydrogenase A), our model accurately focused attention on the macrocyclic core and substrate-mimicking region, both essential for binding. The model precisely identified multiple critical atoms in the macrocyclic portion and key functional groups on the side chains.

Specifically, the model successfully recognized the sulfonamide group that interacts with the catalytic aspartate residue (Asp140), forming crucial hydrogen bonds and ionic interactions. The model correctly highlighted the sulfonamide oxygen atoms that establish multiple interactions with arginine residues (Arg105), which are critical for stabilizing ligand positioning in the active site. The model also accurately identified the importance of the hydroxyl group and adjacent protonated nitrogen atom, which participate in water-mediated hydrogen bond networks with His192 and Glu191. These polar interactions play a crucial role in the binding mechanism of LDHA inhibitors, particularly in mimicking NADH binding site interactions.

Notably, the model appropriately assigned lower importance to nonpolar side chains that play minor roles in binding, such as the cyclohexane portion. This ability to distinguish between critical and non-critical interaction regions demonstrates the model’s accuracy and selectivity. The model’s identification of key residues in the binding pocket, such as Arg105, Asp140, and His192, validates the accuracy of its predictions. These residues are widely recognized as critical targets in LDHA inhibitor design.

For 4N6H (EJ4 with delta-type opioid receptor), our model accurately highlighted the main interacting groups, such as the hydroxyl on the aliphatic ring complex and the adjacent tertiary amine. In the experimental structure, these groups indeed form crucial hydrogen bonds with Aspartate 128 (Asp128).

Importantly, our model did not incorrectly predict the phenol group as participating in protein binding. From our high-resolution prediction image, the phenol hydroxyl group at the top of the molecule is not marked with an orange circle, indicating that our model correctly recognized this region’s limited role in binding. This result aligns with the interaction pattern observed in the actual crystal structure.

Regarding protein residue importance analysis, the model’s interpretability was strong for ligands but relatively weaker for proteins. Occasionally, amino acids distant from the binding site were highlighted, though key residues were also correctly identified. This limitation may be due to the model relying solely on sequence information rather than three-dimensional structure. These findings suggest that incorporating 3D protein data could further enhance interpretability.

The attention-based interpretability of MAGC-DTI provides valuable insights for drug design by revealing molecular interaction patterns learned directly from data. These insights support practical drug discovery and the development of new therapeutics.

## Discussion

In this study, we developed MAGC-DTI, an end-to-end deep learning framework for drug–target interaction prediction that integrates MSAA, AGICA, MPRC, and CDAN to improve cross-modal interaction modeling and robustness under distribution shift. The overall results suggest that MAGC-DTI achieves favorable performance across six benchmark datasets and multiple evaluation settings, while maintaining competitive results in more challenging generalization scenarios, including unseen-drug, unseen-target, and clustering-based cross-domain splits. Although the gains in some settings are modest, the overall trend suggests that the proposed framework is able to capture interaction-relevant patterns that generalize beyond a specific training distribution. This empirical trend is broadly consistent with the design of MAGC-DTI. In particular, MSAA improves protein representation learning by capturing sequence patterns at multiple receptive-field scales, AGICA promotes context-aware cross-modal interaction through a progressively updated shared semantic space, and MPRC helps preserve complementary modality-specific information during fusion and prediction. In addition, CDAN appears to further improve robustness under distribution shift. Taken together, these components provide a plausible explanation for the favorable overall performance of MAGC-DTI under both standard and more rigorous evaluation protocols.

Beyond overall predictive performance, the interpretability analysis offers additional insight into how MAGC-DTI makes its predictions, while also highlighting the current framework’s limitations. In external PDB-based case studies, MAGC-DTI highlighted several ligand regions associated with documented interactions, whereas on the protein side the attention weights were not always concentrated around residues located in the annotated binding pocket. This mismatch likely reflects an important limitation of the current framework, which relies on 1D protein sequences and 2D molecular graphs and therefore does not explicitly encode tertiary structural context or fine-grained spatial determinants of binding. Accordingly, the learned attention patterns should be interpreted as supportive evidence for the model’s predictions rather than as definitive identification of physical binding sites. Nevertheless, the ability of MAGC-DTI to recover documented associations and prioritize plausible candidate interactions suggests that it may still be useful for sequence-based virtual screening and hypothesis generation. Future work may further strengthen the framework by incorporating reliable 3D structural information, such as experimentally resolved structures or high-confidence predicted structures from AlphaFold, and by extending the model toward affinity prediction or related multi-task settings, thereby improving both biological fidelity and practical utility in realistic drug discovery scenarios.

## Methods

### Problem formulation

The drug-target interaction prediction task can be formulated as a binary classification problem. Given a set of drugs $\mathcal {D} = \{d_1, d_2,..., d_m\}$ and a set of protein targets $\mathcal {P} = \{p_1, p_2,..., p_n\}$, our goal is to predict whether a drug-target pair $(d_i, p_j)$ interacts or not. Each drug $d_i$ is represented by its SMILES string, which encodes its molecular structure, while each protein $p_j$ is represented by its amino acid sequence. The prediction task is to learn a function $f: \mathcal {D} \times \mathcal {P} \rightarrow \{0, 1\}$ that maps a drug-target pair to a binary interaction label, where 1 indicates interaction and 0 indicates no interaction.

### Datasets

The MAGC-DTI performance is evaluated on six publicly available datasets: BindingDB^54^, BioSNAP^55^, DrugBank^43^, Human^56^, KIBA^57^, and C.elegans^58^, with test sets composed of unobserved drug-target pairs. Detailed statistics of these six datasets are provided in Supplementary Table S1.

BindingDB^54^ contains experimentally verified binding affinities between small molecule drugs and protein targets, providing quantitative measurements crucial for understanding interaction strengths. BioSNAP^55^ is a balanced dataset derived from DrugBank, where positive DTIs have been validated and negative DTIs were randomly selected from unseen pairs. The DrugBank^43^ dataset combines detailed chemical, pharmacological, and pharmaceutical data with equal numbers of positive and negative samples. Human^56^ contains highly credible negative DTIs verified through bioinformatics tools, significantly reducing false negatives in evaluation. The KIBA^57^ dataset integrates multiple biochemical metrics (Ki, Kd, and IC50) into a unified score system, providing a comprehensive assessment of interaction strengths across kinase inhibitors. *C. elegans*^58^ offers valuable insights into drug mechanisms in model organisms with balanced interaction samples.

### MAGC-DTI architecture

**Fig. 5:**
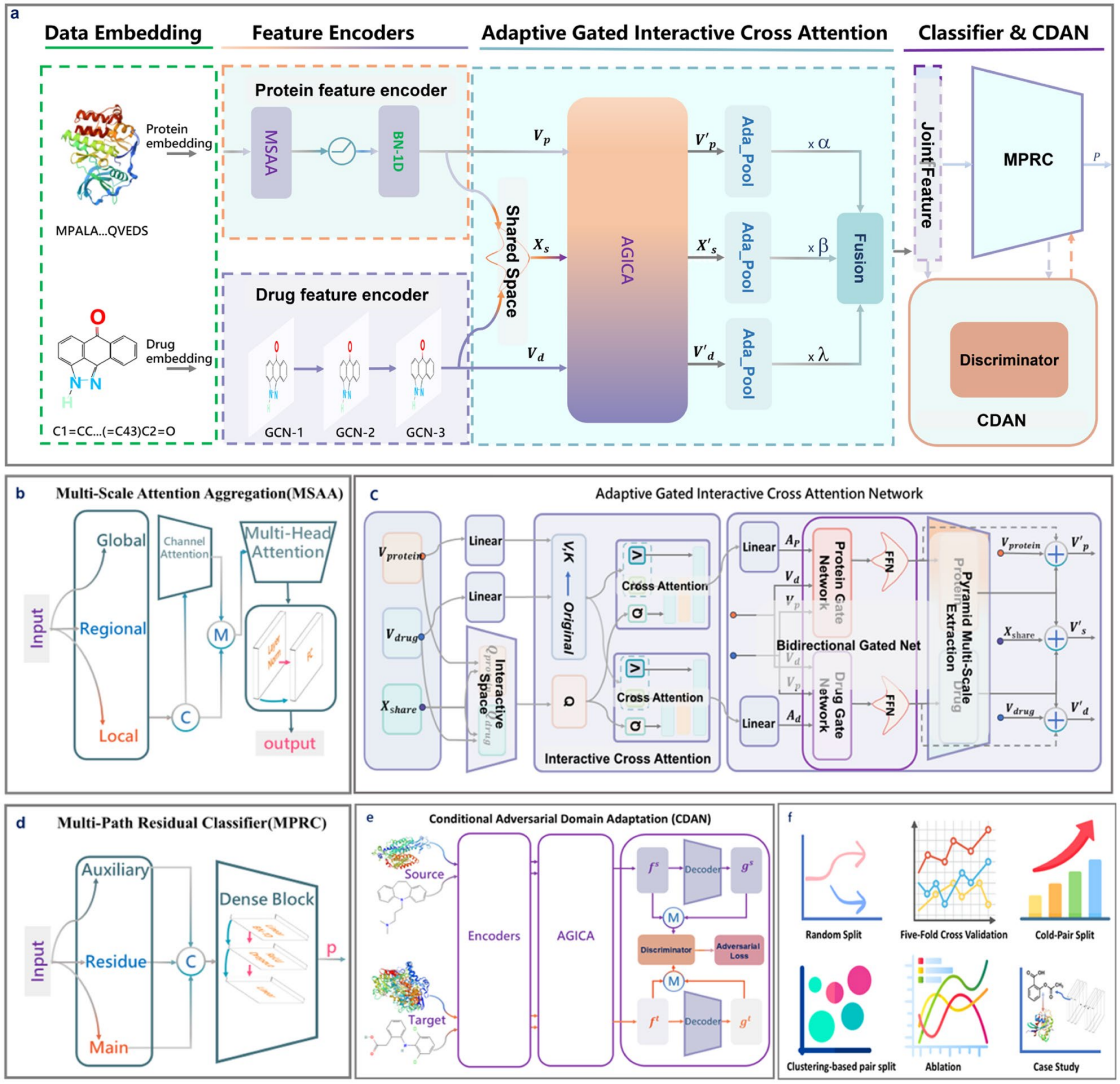
Overview of the MAGC-DTI framework. (**a**) End-to-end pipeline illustrating the sequence of feature extraction, cascaded cross-modal interaction and classification; (**b**) multi-scale protein feature extraction; (**c**) cross-attention between drug and protein representations; (**d**) multi-path residual classifier; (**e**) conditional adversarial domain adaptation schematic; (**f**) summary of training and evaluation protocols. Panels are arranged to reflect model data flow and modular decomposition.

As illustrated in Fig. 5, the MAGC-DTI framework orchestrates drug–target interaction prediction through a progressive three-stage pipeline. First, in the modality-specific encoding stage, drug SMILES are converted into molecular graphs and encoded by a graph convolutional network, while protein sequences are transformed into contextual representations by the Multi-Scale Attention Aggregation (MSAA) module, which captures local-to-global sequence patterns through multi-scale convolution and attention mechanisms. Next, these encoded features enter the Adaptive Gated Interactive Cross Attention (AGICA) module, where a shared latent space and cascaded bidirectional interaction mechanism are used to iteratively propagate and refine cross-modal signals between drug and protein representations. Finally, the refined modality-specific and shared features are integrated by the Multi-Path Residual Classifier (MPRC), which performs residual feature fusion and hierarchical decoding to produce the final interaction probability. In clustering-based cross-domain settings, Conditional Adversarial Domain Adaptation (CDAN) is further introduced to enhance the robustness of the learned representation against domain shift.

#### Drug feature extraction with molecular graph convolution

In the drug feature encoder, each compound is first converted from its SMILES representation into a molecular graph, following the general graph-based drug feature extraction strategy in Ref.^32^. Specifically, given a drug compound *d*, we represent it as a graph $G_d=(V_d,E_d)$ by processing its SMILES string^59^, where $V_d$ and $E_d$ denote the sets of atoms and chemical bonds, respectively. Atom-level features are initialized from their chemical properties using the DGL-LifeSci package^60^, including atom type, hybridization state, formal charge, and aromaticity. Since different drug molecules contain different numbers of atoms, zero-padding is applied to obtain graph representations with consistent dimensions for batch training. A multi-layer graph convolutional network (GCN) is then used to encode the molecular graph by iteratively aggregating neighborhood information:

1 $$\begin{aligned} H_d^{(l+1)}=\phi \!\left( f_{\textrm{aggregate}}\!\left( H_d^{(l)},A_d\right) W_d^{(l)}\right) , \end{aligned}$$

where $H_d^{(l)}$ denotes the atom feature matrix at layer *l*, $A_d$ is the adjacency matrix of the molecular graph, $W_d^{(l)}$ is a learnable weight matrix, and $\phi (\cdot )$ denotes a nonlinear activation function. Through iterative neighborhood aggregation, the model captures local chemical environments and higher-order topological patterns. After the final graph convolution layer, a pooling operation is applied to obtain a fixed-length drug representation, denoted by $V_d$, which is used for subsequent cross-modal interaction modeling.

#### Multi-scale attention aggregation

In the protein feature encoder, the amino acid sequence is first mapped into a trainable embedding space and then processed by the Multi-Scale Attention Aggregation (MSAA) module. As illustrated in Fig. 5b, MSAA combines multi-scale convolutional feature extraction and adaptive attention weighting to capture hierarchical sequence patterns at different receptive-field scales.

Given a protein sequence *p*, we first obtain its embedding representation $X_p=\textrm{Embedding}(p)$. MSAA then extracts features through three parallel convolution branches with different kernel sizes to model local, regional, and broader contextual patterns. These multi-scale features are concatenated and adaptively reweighted:

2 $$\begin{aligned} X_p^{\textrm{fused}}= \left[ X_p^{(1)};X_p^{(3)};X_p^{(5)}\right] \odot \sigma \!\left( \textrm{FC}\!\left( \textrm{AdaptiveAvgPool2d}\!\left( [X_p^{(1)};X_p^{(3)};X_p^{(5)}]\right) \right) \right) , \end{aligned}$$

where $X_p^{(1)}$, $X_p^{(3)}$, and $X_p^{(5)}$ denote the outputs of the three convolution branches, and $\sigma (\cdot )$ denotes the sigmoid activation function. This adaptive weighting allows the model to emphasize the most informative scale for each protein.

To further model long-range dependencies, the fused features are reshaped and refined by multi-head self-attention with residual normalization:

3 $$\begin{aligned} V_p=\textrm{FeatureAlign}\!\left( \textrm{LayerNorm}\!\left( \textrm{MHA}(\widetilde{X}_p,\widetilde{X}_p,\widetilde{X}_p)+\widetilde{X}_p\right) \right) , \end{aligned}$$

where $\widetilde{X}_p$ denotes the reshaped multi-scale feature tensor and $\textrm{MHA}(\cdot )$ denotes multi-head self-attention. The resulting representation $V_p$ encodes both multi-scale sequence information and long-range contextual dependencies for downstream interaction learning.

#### Adaptive gated interactive cross attention

Given the encoded protein feature $V_p$ and drug feature $V_d$, the Adaptive Gated Interactive Cross Attention (AGICA) module performs cascaded bidirectional cross-modal interaction to progressively refine modality-specific representations. As illustrated in Fig. 5c, AGICA also maintains a shared feature space to mediate information exchange between the two modalities.

At the beginning of AGICA, a shared feature $X_s^{(0)}$ is initialized from the concatenated protein and drug features, and the encoded features are used as the initial modality-specific inputs. In each cascaded interaction layer, residual feature transformation is first applied separately to the two modalities, after which shared-space-guided interaction centers are constructed and used to perform bidirectional cross-attention. The cross-modal attention outputs are denoted by

4 $$\begin{aligned} A_{d\leftarrow p}^{(i)}=\textrm{MultiHeadAttn}\!\left( Q_d^{(i)},K_p^{(i)},V_p^{(i)}\right) , \qquad A_{p\leftarrow d}^{(i)}=\textrm{MultiHeadAttn}\!\left( Q_p^{(i)},K_d^{(i)},V_d^{(i)}\right) . \end{aligned}$$

To selectively integrate the exchanged information, AGICA introduces an adaptive gating mechanism:

5 $$\begin{aligned} \tilde{x}_m^{(i)}=\bar{x}_m^{(i)}\odot (1-G_m^{(i)})+O_m^{(i)}\odot G_m^{(i)}, \qquad m\in \{p,d\}, \end{aligned}$$

where $\bar{x}_m^{(i)}$ denotes the transformed modality-specific feature, $O_m^{(i)}$ denotes the projected cross-attention output, and $G_m^{(i)}$ denotes the learned gate. The gated features are then refined by a feed-forward block and enhanced by a global contextual branch based on pyramid pooling. Meanwhile, the shared feature is updated from the global contextual features of both modalities:

6 $$\begin{aligned} x_s^{(i)}=\textrm{BN}_s\!\left( \textrm{Conv}_{1\times 1}\!\left( [\textrm{Global}_p^{(i)};\textrm{Global}_d^{(i)}]\right) \right) +x_s^{(i-1)}. \end{aligned}$$

After *N* cascaded layers, AGICA outputs the refined protein, shared, and drug features, denoted by $V_p'$, $X_s'$, and $V_d'$, respectively. Through this cascaded design, AGICA progressively refines both modality-specific and shared representations and enables more effective bidirectional cross-modal information exchange.

#### Multi-path residual classifier

The Multi-Path Residual Classifier (MPRC) aggregates the refined protein, shared, and drug features to predict the interaction probability. As shown in Fig. 5d, the three branches are first compressed into fixed-length vectors by adaptive pooling, yielding pooled features $z_p$, $z_s$, and $z_d$. These vectors are concatenated and fused into a joint feature:

7 $$\begin{aligned} f_{\textrm{joint}}=\textrm{LayerNorm}\!\left( \textrm{Linear}_{\textrm{fusion}}([z_p;z_s;z_d])\right) . \end{aligned}$$

To preserve complementary information, MPRC further processes the fused feature through two parallel pathways and then integrates them with the original input through residual fusion. The final interaction probability is computed as

8 $$\begin{aligned} \hat{y}=\sigma \!\left( \textrm{MLP}(f_{\textrm{joint}})\right) , \end{aligned}$$

where $\hat{y}$ denotes the predicted interaction probability.

#### Cross-domain adaptation with CDAN

To improve robustness under distribution shift, we further integrate Conditional Adversarial Domain Adaptation (CDAN)^61^ on top of MAGC-DTI. CDAN is enabled only in clustering-based cross-domain evaluation and is disabled in the other settings. Let *F(· )* denote the feature extractor that outputs the joint feature $f_{\textrm{joint}}$, and let *G(· )* denote the classifier that produces a two-class probability vector $g\in \mathbb {R}^{2}$.

Following CDAN, a multilinear conditioning representation is constructed as

9 $$\begin{aligned} h=\operatorname {FLATTEN}(f_{\textrm{joint}}\otimes g), \end{aligned}$$

where *⊗* denotes the outer product. A domain discriminator is then trained adversarially to align source and target feature distributions. The overall optimization objective is

10 $$\begin{aligned} \max _D\min _{F,G}\ \mathcal {L}_{\textrm{cls}}(F,G)-\omega \,\mathcal {L}_{\textrm{adv}}(F,G,D), \qquad \omega >0, \end{aligned}$$

where $\mathcal {L}_{\textrm{cls}}$ and $\mathcal {L}_{\textrm{adv}}$ denote the supervised classification loss and adversarial domain adaptation loss, respectively. Only the source and target training partitions are used for domain alignment; validation and test sets are never involved in adversarial training.

### Training and optimization

We train MAGC-DTI using binary cross-entropy loss:

11 $$\begin{aligned} \mathcal {L} = -\frac{1}{N} \sum _{i=1}^{N} [y_i \log (\hat{y}_i) + (1-y_i) \log (1-\hat{y}_i)] \end{aligned}$$

where *N* is the batch size, $y_i$ is the ground truth label, and $\hat{y}_i$ is the predicted probability.

### Evaluation scenarios

To comprehensively assess model generalizability and robustness, we conducted experiments under three data partitioning strategies plus a clustering-based evaluation approach:

S1: Random split. Each dataset was randomly divided into training, validation, and test sets at a ratio of 7:1:2. Under this setting, the distributions of the sizes of drugs (i.e., the number of atoms) and targets (i.e., the number of amino acids) in the validation/training/test sets were described in the form of categorical scatter plots. The results are available at: https://github.com/wbw176-netizen/MAGC-DTI/tree/main/Distributions.

S2: 5-fold cross-validation. All drug-target pairs were randomly partitioned into five approximately equal subsets, where in each fold, 80% of the drug-target pairs were randomly selected for training while the remaining 20% served as the test set.

S3: Cold pair split. Training, validation, and test sets were constructed to ensure that specific drug-target pairs in the test set do not appear in the training set, while individual drugs and targets may still be present in both sets but in different combinations. This evaluates the model’s ability to predict novel interactions between known drugs and targets.

S4: Unseen drug and unseen target settings. To further assess cold-start generalization at the entity level, we additionally constructed unseen drug and unseen target settings. In the unseen drug setting, drugs appearing in the test set were excluded from the training set. In the unseen target setting, targets appearing in the test set were excluded from the training set. These settings evaluate the model’s ability to generalize to previously unseen drugs or targets.

### Evaluation metrics

We evaluate MAGC-DTI using the area under the receiver operating characteristic curve (AUROC) and the area under the precision–recall curve (AUPRC). All experiments are implemented in PyTorch and conducted on NVIDIA RTX 4060 GPUs.

### Implementation details

We first determined a stable hyperparameter configuration through preliminary experiments on the Human dataset based on validation performance, and then fixed the same configuration for all remaining datasets unless otherwise specified. This strategy avoids dataset-specific hyperparameter tuning and enables a more consistent evaluation across benchmarks. For optimization, we used the Adam optimizer with an initial learning rate of $1\times 10^{-4}$ and applied a decay factor of 0.5 when validation performance plateaued. To mitigate overfitting, we employed dropout, weight decay, and early stopping based on validation performance. For reproducibility, all reported results are presented as mean and standard deviation over multiple runs with different random seeds. The main training hyperparameters were set as follows: MAX_EPOCH=100, BATCH_SIZE=64, LR=1e-4, LR_DECAY=0.5, WEIGHT_DECAY=1e-5, NODE_IN_EMBEDDING=128, EMBEDDING_DIM=128, NUM_CASCADES=2, NUM_HEAD=4, and HIDDEN_DIM=768.

## Supplementary Information

Supplementary Information.

## Data Availability

The data and code that support the findings of this study are available at: https://github.com/wbw176-netizen/MAGC-DTI.
