# Supplementary material for: MAGC-DTI: modality-shared space and adaptive gated interactive cross-attention for drug–target interaction prediction
Source: Sci Rep. 2026 Jun 4;16:25426. doi: 10.1038/s41598-026-50141-w (PMC13473133; doi:10.1038/s41598-026-50141-w)
Supplement: Supplementary file 1 [file 41598_2026_50141_MOESM1_ESM.pdf]

Supplementary Information for:
MAGC-DTI: Modality-Shared Space and Adaptive Gated Interactive Cross-Attention for Drug–Target Interaction Prediction

Bowen Wang[1,2,+], Xiaolan Xie[1,*], Haitao Zou[2,+], and Shaoliang Peng[2,*]

[1]College of Computer Science and Engineering, Guilin University of Technology, Guilin, Guangxi 541006, China
[2]College of Computer Science and Electronic Engineering, Hunan University, Changsha, Hunan 410082, China
[*]Correspondence: slpeng@hnu.edu.cn (S.P.); xie_xiao_lan@foxmail.com (X.X.)
[+]These authors contributed equally to this work.

Supplementary Tables

Supplementary Table S1: Statistics of Six DTI Datasets

**Dataset**	**Drugs**	**Proteins**	**Positive**	**Negative**	**Total**
BindingDB	14,643	2,623	20,674	28,525	49,199
BioSNAP	4,510	2,181	13,634	13,830	27,464
DrugBank	4,254	6,645	17,511	17,511	35,022
KIBA	2,068	225	22,154	94,196	116,350
Human	2,726	2,001	2,633	3,364	5,997
C.elegans	1,767	1,876	3,893	3,893	7,786

Supplementary Table S2: Effect of replacing the default molecular and protein encoders with PLMs (ChemBERTa and ESM-2) under the random split setting.

**Dataset**	**MAGC-DTI**		**MAGC-DTI + PLMs**		**Gain (%)**	
	**AUROC**	**AUPRC**	**AUROC**	**AUPRC**	**AUROC**	**AUPRC**
DrugBank	0.9075±0.0010	0.9147±0.0020	0.9402±0.0020	0.9389±0.0010	3.27	2.42
BioSNAP	0.9286±0.0023	0.9350±0.0040	0.9419±0.0020	0.9409±0.0030	1.33	0.59

Supplementary Table S3: Performance comparison on BindingDB, BioSNAP, and DrugBank datasets under 5-fold cross-validation.

Method	BindingDB		BioSNAP		DrugBank	
	**AUROC**	**AUPRC**	**AUROC**	**AUPRC**	**AUROC**	**AUPRC**
MolTrans	0.9418±0.0049	0.9238±0.0102	0.8895±0.0072	0.8949±0.0041	0.8709±0.0080	0.8790±0.0092
Drug-BAN	0.9650±0.0010	0.9530±0.0020	0.9110±0.0040	0.9080±0.0030	0.8850±0.0030	0.8790±0.0040
BINDTI	0.9655±0.0036	0.9519±0.0045	0.9046±0.0028	0.9061±0.0041	0.8881±0.0066	0.8884±0.0097
MADTI	0.9612±0.0025	0.9513±0.0030	0.9102±0.0016	0.9010±0.0028	0.8820±0.0030	0.8820±0.0040
WDGBANDTI	0.9643±0.0015	0.9522±0.0038	0.9117±0.0032	0.9169±0.0022	0.8856±0.0020	0.8892±0.0030
ASCS-DTI	0.9639±0.0038	0.9488±0.0058	0.9149±0.0047	0.9158±0.0060	0.8818±0.0031	0.8850±0.0037
CF-DTI	0.9618±0.0046	0.9512±0.0050	0.9134±0.0030	0.9150±0.0040	0.8821±0.0048	0.8870±0.0052
**MAGC-DTI**	**0.9663±0.0018**	**0.9562±0.0027**	**0.9205±0.0015**	**0.9209±0.0027**	**0.9101±0.0010**	**0.9124±0.0030**

Supplementary Table S4: Performance comparison on Human, KIBA, and C.elegans datasets under 5-fold cross-validation.

Method	Human		KIBA		C.elegans	
	**AUROC**	**AUPRC**	**AUROC**	**AUPRC**	**AUROC**	**AUPRC**
MolTrans	0.9809±0.0078	0.9799±0.0063	0.9132±0.0020	0.7949±0.0016	0.9820±0.0030	0.9820±0.0030
Drug-BAN	0.9839±0.0023	0.9816±0.0035	0.9208±0.0042	0.7833±0.0101	0.9910±0.0015	0.9895±0.0043
BINDTI	0.9794±0.0036	0.9753±0.0102	0.9205±0.0030	0.7992±0.0044	0.9922±0.0010	0.9913±0.0020
MADTI	0.9837±0.0012	0.9801±0.0027	0.9199±0.0020	0.7883±0.0050	0.9886±0.0020	0.9875±0.0030
WDGBANDTI	0.9826±0.0017	0.9789±0.0022	0.9258±0.0018	0.7949±0.0054	0.9914±0.0027	0.9913±0.0033
ASCS-DTI	0.9832±0.0035	0.9814±0.0043	0.9218±0.0025	0.7939±0.0030	0.9884±0.0028	0.9913±0.0030
CF-DTI	0.9855±0.0028	0.9811±0.0032	0.9262±0.0034	0.7954±0.0045	0.9910±0.0018	0.9913±0.0019
**MAGC-DTI**	**0.9880±0.0022**	**0.9847±0.0025**	**0.9331±0.0013**	**0.8125±0.0030**	**0.9953±0.0020**	**0.9959±0.0016**

Supplementary Table S5: Statistical tests on six benchmark datasets under S1 and 5-fold cross-validation.

Dataset	Method	S1			5-fold cross-validation		
		**Difference**	t	p	**Difference**	t	p
BindingDB	BINDTI	0.0094	9.6503	< 0.001	0.0008	0.6163	0.5710
	WDGBANDTI	0.0094	9.3533	< 0.001	0.0020	2.9814	0.0407
	ASCS-DTI	0.0051	3.5482	0.0238	0.0024	1.7437	0.1562
	CF-DTI	0.0055	5.6076	0.0050	0.0045	2.6458	0.0572
BioSNAP	BINDTI	0.0247	15.6179	< 0.001	0.0159	15.8211	< 0.001
	WDGBANDTI	0.0146	13.3168	< 0.001	0.0088	7.5851	0.0016
	ASCS-DTI	0.0122	6.7179	0.0026	0.0056	3.1423	0.0348
	CF-DTI	0.0113	13.0135	< 0.001	0.0071	6.5640	0.0028
DrugBank	BINDTI	0.0256	17.3544	< 0.001	0.0220	8.1270	0.0012
	WDGBANDTI	0.0218	30.2312	< 0.001	0.0245	33.9754	< 0.001
	ASCS-DTI	0.0244	33.8367	< 0.001	0.0283	24.1080	< 0.001
	CF-DTI	0.0215	19.0035	< 0.001	0.0280	14.6438	< 0.001
Human	BINDTI	0.0101	6.3904	0.0031	0.0086	6.6765	0.0026
	WDGBANDTI	0.0091	20.8330	< 0.001	0.0054	6.7061	0.0026
	ASCS-DTI	0.0074	4.6820	0.0094	0.0048	3.8308	0.0186
	CF-DTI	0.0042	5.7691	0.0045	0.0025	2.4310	0.0719
KIBA	BINDTI	0.0225	13.6376	< 0.001	0.0126	11.4926	< 0.001
	WDGBANDTI	0.0131	7.7022	0.0015	0.0073	11.2056	< 0.001
	ASCS-DTI	0.0133	7.8694	0.0014	0.0113	12.5711	< 0.001
	CF-DTI	0.0072	4.3411	0.0122	0.0069	5.4734	0.0054
C.elegans	BINDTI	0.0087	12.1586	< 0.001	0.0031	4.2989	0.0127
	WDGBANDTI	0.0044	9.8387	< 0.001	0.0039	3.9763	0.0165
	ASCS-DTI	0.0070	6.3271	0.0032	0.0069	6.8187	0.0024
	CF-DTI	0.0034	4.7516	0.0090	0.0043	5.6268	0.0049

Supplementary Table S6: Performance comparison on the BioSNAP dataset under unseen drug and unseen target settings.

Method	Unseen drug (AUROC)	Unseen target (AUROC)
MolTrans	0.8530±0.0110	0.7489±0.0290
Drug-BAN	0.8672±0.0050	0.7620±0.0160
BINDTI	0.8650±0.0011	0.6728±0.0100
MADTI	0.8683±0.0040	0.7371±0.0240
WDGBANDTI	0.8613±0.0060	0.7570±0.0190
ASCS-DTI	0.8579±0.0040	0.7532±0.0050
CF-DTI	0.8660±0.0060	0.7671±0.0290
**MAGC-DTI**	**0.8735±0.0050**	**0.7720±0.0200**

Supplementary Table S7: Ablation Study: Impact of AGICA Module on Model Performance

**Method**	**BindingDB**		**BioSNAP**		**DrugBank**	
	**AUROC**	**AUPRC**	**AUROC**	**AUPRC**	**AUROC**	**AUPRC**
MAGC-DTI	0.9701±0.0027	0.9613±0.0010	0.9286±0.0023	0.9350±0.0040	0.9075±0.0010	0.9147±0.0020
MAGC-DTI w/o AGICA	0.9581±0.0028	0.9442±0.0051	0.9024±0.0003	0.9088±0.0016	0.8712±0.0014	0.8782±0.0062
Performance Drop	1.2%±0.3%	1.7%±0.4%	2.8%±0.2%	2.8%±0.3%	4.0%±0.2%	4.0%±0.5%
**Method**	**Human**		**KIBA**		**C.elegans**	
	**AUROC**	**AUPRC**	**AUROC**	**AUPRC**	**AUROC**	**AUPRC**
MAGC-DTI	0.9914±0.0009	0.9878±0.0009	0.9365±0.0046	0.8213±0.0014	0.9960±0.0012	0.9966±0.0005
MAGC-DTI w/o AGICA	0.9856±0.0010	0.9791±0.0020	0.9301±0.0020	0.8097±0.0010	0.9912±0.0010	0.9924±0.0013
Performance Drop	0.6%±0.1%	0.9%±0.2%	0.7%±0.3%	1.4%±0.2%	0.5%±0.1%	0.4%±0.1%

Supplementary Table S8: Ablation Study: Impact of MSAA Mechanism on Model Performance

**Method**	**BindingDB**		**BioSNAP**		**DrugBank**	
	**AUROC**	**AUPRC**	**AUROC**	**AUPRC**	**AUROC**	**AUPRC**
MAGC-DTI	0.9701±0.0027	0.9613±0.0010	0.9286±0.0023	0.9350±0.0040	0.9075±0.0010	0.9147±0.0020
MAGC-DTI w/o MSAA	0.9613±0.0020	0.9482±0.0012	0.9084±0.0014	0.9128±0.0028	0.8872±0.0010	0.8811±0.0030
Performance Drop	0.9%±0.3%	1.4%±0.2%	2.2%±0.2%	2.4%±0.4%	2.2%±0.1%	3.7%±0.3%
**Method**	**Human**		**KIBA**		**C.elegans**	
	**AUROC**	**AUPRC**	**AUROC**	**AUPRC**	**AUROC**	**AUPRC**
MAGC-DTI	0.9914±0.0009	0.9878±0.0009	0.9365±0.0046	0.8213±0.0014	0.9960±0.0012	0.9966±0.0005
MAGC-DTI w/o MSAA	0.9843±0.0010	0.9815±0.0020	0.9258±0.0010	0.8016±0.0010	0.9923±0.0012	0.9956±0.0008
Performance Drop	0.7%±0.1%	0.6%±0.2%	1.1%±0.3%	2.4%±0.1%	0.4%±0.1%	0.1%±0.1%

Supplementary Table S9: Ablation Study: Impact of Multi-Path Residual Classifier on Model Performance

**Method**	**BindingDB**		**BioSNAP**		**DrugBank**	
	**AUROC**	**AUPRC**	**AUROC**	**AUPRC**	**AUROC**	**AUPRC**
MAGC-DTI	0.9701±0.0027	0.9613±0.0010	0.9286±0.0023	0.9350±0.0040	0.9075±0.0010	0.9147±0.0020
MAGC-DTI w/o MPRC	0.9629±0.0020	0.9511±0.0003	0.9124±0.0067	0.9202±0.0065	0.8882±0.0008	0.8968±0.0009
Performance Drop	0.7%±0.3%	1.1%±0.1%	1.7%±0.5%	1.6%±0.6%	2.1%±0.1%	2.0%±0.2%
**Method**	**Human**		**KIBA**		**C.elegans**	
	**AUROC**	**AUPRC**	**AUROC**	**AUPRC**	**AUROC**	**AUPRC**
MAGC-DTI	0.9914±0.0009	0.9878±0.0009	0.9365±0.0046	0.8213±0.0014	0.9960±0.0012	0.9966±0.0005
MAGC-DTI w/o MPRC	0.9852±0.0022	0.9823±0.0028	0.9330±0.0004	0.8158±0.0022	0.9934±0.0010	0.9943±0.0010
Performance Drop	0.6%±0.2%	0.6%±0.3%	0.4%±0.3%	0.7%±0.2%	0.3%±0.1%	0.2%±0.1%

Supplementary Table S10: Ablation Study: Impact of GCN Module on Model Performance

**Method**	**BindingDB**		**BioSNAP**		**DrugBank**	
	**AUROC**	**AUPRC**	**AUROC**	**AUPRC**	**AUROC**	**AUPRC**
MAGC-DTI	0.9701±0.0027	0.9613±0.0010	0.9286±0.0023	0.9350±0.0040	0.9075±0.0010	0.9147±0.0020
MAGC-DTI w/o GCN	0.9623±0.002	0.9514±0.001	0.9152±0.0034	0.9178±0.0013	0.8907±0.001	0.8971±0.001
Performance Drop	0.8%±0.3%	1.0%±0.1%	1.4%±0.3%	1.8%±0.4%	1.9%±0.1%	1.9%±0.2%
**Method**	**Human**		**KIBA**		**C.elegans**	
	**AUROC**	**AUPRC**	**AUROC**	**AUPRC**	**AUROC**	**AUPRC**
MAGC-DTI	0.9914±0.0009	0.9878±0.0009	0.9365±0.0046	0.8213±0.0014	0.9960±0.0012	0.9966±0.0005
MAGC-DTI w/o GCN	0.9855±0.002	0.9815±0.0012	0.9308±0.001	0.8101±0.001	0.9887±0.002	0.9890±0.001
Performance Drop	0.6%±0.2%	0.6%±0.1%	0.6%±0.4%	1.4%±0.1%	0.7%±0.2%	0.8%±0.1%

Supplementary Table S11: Effect of the CDAN module under the cross-domain setting.

**Method**	**BindingDB**		**BioSNAP**	
	**AUROC**	**AUPRC**	**AUROC**	**AUPRC**
MAGC-DTI	**0.6832±0.0050**	**0.5982±0.0040**	**0.7152±0.0030**	**0.7623±0.0040**
w/o CDAN	0.6317±0.0360	0.5681±0.0200	0.6986±0.0260	0.7330±0.0230

Supplementary Table S12: Top 10 Drug Candidates Predicted to Interact with H1R and ADRA1C by MAGC-DTI

**Rank**	**H1R (P35367)**		**ADRA1C (P35348)**	
	**DrugBank ID**	**Score**	**DrugBank ID**	**Score**
1	DB00751	0.999996	DB00321	0.999995
2	DB00408	0.999994	DB01614	0.999992
3	DB01615	0.999990	DB01238	0.999990
4	DB00363	0.999990	DB01175	0.999989
5	DB01238	0.999988	DB09167	0.999988
6	DB00366	0.999982	DB01069	0.999988
7	DB00543	0.999982	DB00243	0.999986
8	DB00246	0.999982	DB00408	0.999986
9	DB00215	0.999976	DB00751	0.999985
10	DB01175	0.999976	DB01624	0.999985
